# Screening for Hearing Impairment in Older Adults by Smartphone-Based Audiometry, Self-Perception, HHIE Screening Questionnaire, and Free-Field Voice Test: Comparative Evaluation of the Screening Accuracy With Standard Pure-Tone Audiometry

**DOI:** 10.2196/17213

**Published:** 2020-10-27

**Authors:** Lok Yee Joyce Li, Shin-Yi Wang, Cheng-Jung Wu, Cheng-Yu Tsai, Te-Fang Wu, Yaoh-Shiang Lin

**Affiliations:** 1 Department of Medicine, Shin Kong Wu-Ho-Su Memorial Hospital Taipei Taiwan; 2 Department of Otolaryngology, Shuang Ho Hospital, Taipei Medical University New Taipei City Taiwan; 3 Department of Otolaryngology, School of Medicine, College of Medicine, Taipei Medical University Taipei Taiwan; 4 National Taiwan University Hospital Hsin-chu Branch Hsin-chu Taiwan; 5 PhD Degree Program of Biomedical Science and Engineering, National Chiao Tung University Hsinchu Taiwan; 6 Department of Otolaryngology-Head And Neck Surgery, Tri-Service General Hospital Taipei Taiwan; 7 Department of Civil and Environmental Engineering, Imperial College London London United Kingdom; 8 Department of Otolaryngology - Head And Neck Surgery, Kaohsiung Veterans General Hospital Kaohsiung Taiwan

**Keywords:** hearing impairment, self-perception, HHIE-S questionnaire, free-field voice test, mobile phone, audiometry, mobile health

## Abstract

**Background:**

Hearing impairment is the most frequent sensory deficit in humans, affecting more than 360 million people worldwide. In fact, hearing impairment is not merely a health problem, but it also has a great impact on the educational performance, economic income, and quality of life. Hearing impairment is therefore an important social concern.

**Objective:**

We aimed to evaluate and compare the accuracy of self-perception, Hearing Handicap Inventory for the Elderly-Screening (HHIE-S) questionnaire, free-field voice test, and smartphone-based audiometry as tests for screening moderate hearing impairment in older adults in China.

**Methods:**

In this study, 41 patients were recruited through a single otology practice. All patients were older than 65 years. Patients with otorrhea and cognitive impairment were excluded. Moderate hearing impairment was defined as mean hearing thresholds at 500, 1000, 2000, and 4000 Hz >40 dB hearing loss (pure-tone average > 40 dB hearing loss). All patients completed 5 hearing tests, namely, the self-perception test, HHIE-S questionnaire test, free-field voice test, smartphone-based audiometry test, and standard pure-tone audiometry by the same audiologist. We compared the results of these tests to the standard audiogram in the better-hearing ear.

**Results:**

The sensitivity and the specificity of the self-perception test were 0.58 (95% CI 0.29-0.84) and 0.34 (95% CI 0.19-0.54), respectively. The sensitivity and the specificity of the HHIE-S questionnaire test were 0.67 (95% CI 0.35-0.89) and 0.31 (95% CI 0.316-0.51), respectively. The sensitivity and the specificity of the free-field voice test were 0.83 (95% CI 0.51-0.97) and 0.41 (95% CI 0.24-0.61), respectively. The sensitivity and the specificity of the smartphone-based audiometry test were 0.92 (95% CI 0.60-0.99) and 0.76 (95% CI 0.56-0.89), respectively. Smartphone-based audiometry correctly diagnosed the presence of hearing loss with high sensitivity and high specificity.

**Conclusions:**

Smartphone-based audiometry may be a dependable screening test to rule out moderate hearing impairment in the older population.

## Introduction

Sensory deficit is defined as a condition wherein any one of the senses, that is, sight, hearing, touch, taste, or smell is no longer functioning normally. Based on the available data, the 2 most commonly encountered sensory impairments are blindness and deafness [1]. Hearing impairment is one of the most frequent sensory deficits in human beings, and it has a profound effect on the life of the affected persons, their families, and the society as a whole. Hearing impairment is actually not just a health problem, because it affects the educational opportunities, the economic situation, and the quality of life of individuals with these impairments. Hearing impairment affects more than 5% of the world’s population. In 2012, the World Health Organization released new estimates on the magnitude of disabling hearing loss [2]. The estimates are based on a review of 42 population-based studies carried out up to 2010 [3]. Based on these studies, the World Health Organization estimated that there are 360 million persons in the world with disabling hearing loss. Approximately 328 million of these are adults and 32 million of these are children. About one-third of the persons older than 65 years are affected by hearing impairment [2]. The prevalence of hearing impairment in adults over 65 years is the highest in limited-income countries. However, over 50% of the causes for hearing impairment are preventable [4].

A large number of cases of hearing loss are preventable and many can be treated effectively and immediately. People with hearing losses due to other causes that cannot be treated effectively can be rehabilitated through various available measures, and the integration of such people into the society can be improved [1]. However, limited-income countries face many problems in order to achieve the global aim of preventing and rehabilitating hearing impairments in older adults [5]. The first challenge in these limited-income countries is that medical equipment are limited. Advanced diagnostic tools and standard audiometry tests are quite limited. Due to the limited medical supplies, primary health care professionals face difficult triage decisions such as “Who gets to see an audiologist and undergo standard audiometry tests and who will only see a nurse or a community health educator and follow up?” The second challenge in these limited-income countries is the high incidence of conditions that cause hearing loss compared to that in industrialized countries [6]. Chronic otitis media infections constitute the major disease burden in low-income countries. The third challenge is that human resources are also limited. In these countries, it is common for only 1 physician to see to more than 100,000 people. Besides, trained audiologists in these countries are lacking compared to those in industrialized countries.

The gold standard for diagnosing hearing impairment is the standard audiogram. However, there can be financial or geographic obstacles to receiving a timely audiogram test [5]. These problems may lead to delays in the diagnosis of hearing impairment. Delay in the diagnosis of hearing impairment may lead to delay in treatment, which is considered to be associated with low rates of hearing recovery. Owing to the above obstacles, we aimed to develop a simple, rapid, easily applicable, and cost-effective hearing test for the assessment of hearing conditions in low-income countries. With the development of mobile health technology, smartphone-based hearing tests have been developed as screening tools to identify patients with hearing loss. In this study, we evaluated smartphone-based audiometry as a test for screening moderate hearing impairment in older adults and we aimed to validate this test against standard pure-tone audiometry. This study also compared the usefulness of self-perception, the Hearing Handicap Inventory for the Elderly-Screening (HHIE-S) questionnaire, and free-field voice test to screen for moderate hearing impairment in the older adults in China. In this paper, we discuss the accuracy of the hearing-loss screening methods in older adults, including self-perception, HHIE-S questionnaire, free-field voice test, and smartphone-based audiometry.

## Methods

### Patient Selection

In this study, 41 patients were recruited through a single otology practice in the Kaohsiung Veterans General Hospital. All included patients were older than 65 years. All patients were fluent in Chinese and were able to read and write in Chinese. Patients with active otorrhea, cognitive impairment, Parkinson disease, clinically diagnosed dementia, and hand action tremor were excluded. Patients were excluded if they used hearing aids or received a standard pure-tone audiogram evaluation in the prior 24 months or were unable to complete questionnaires. Patients with conductive hearing loss based on standard pure-tone audiometry hearing test were also excluded as it would have made interpretation of an air-conduction smartphone-based audiometry hearing test impossible. The 5 hearing tests were performed for each patient in a randomized order during a single visit. Free-field voice test, smartphone-based audiometry test, and standard pure-tone audiometry were performed in the same soundproof room with average ambient noise level varying between 38 dBA and 39 dBA-weighted sound pressure level. Informed consent was obtained from each of the patients in this study.

### Study Design

All patients completed 5 hearing evaluations, that is, the self-perception test, HHIE-S questionnaire test, free-field voice test, smartphone-based audiometry test, and a standard pure-tone audiometry test. All the hearing tests were conducted by the same audiologist. We compared the results of the self-perception test, HHIE-S questionnaire test, free-field voice test, and smartphone-based audiometry test to those of the standard pure-tone audiogram.

### Screening Strategies

#### Self-Perception Hearing Screening Test

The self-perception hearing screening test was examined using a single question. The question was “Do you have a hearing problem now?” (您現在有聽力問題嗎?) This sentence was used as the subjective criterion of hearing impairment [7]. A yes or an equivalent response to this question was considered as a positive screen for hearing impairment [7]. Participating patients were asked to respond as yes or no. Patients with the answer “yes” were considered to have screened positive for moderate hearing loss [8].

#### HHIE-S Questionnaire

**Figure 1:**
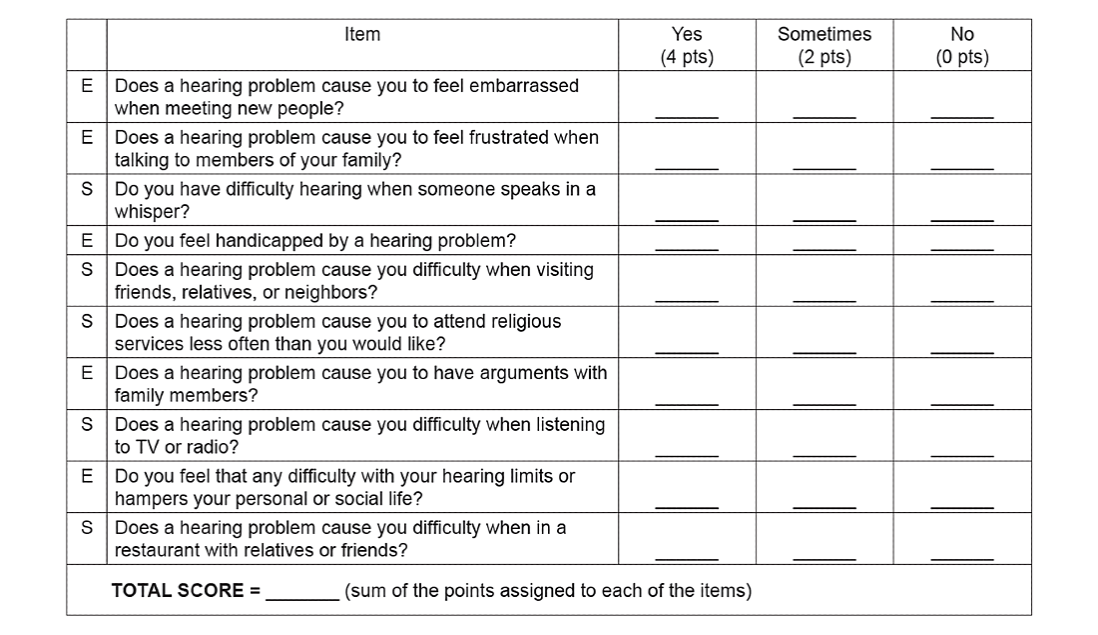
Hearing Handicap Inventory for the Elderly Screening Version.

The HHIE-S questionnaire is a 10-item, self-administered questionnaire developed to assess how an older patient perceives the social and emotional effects of hearing impairment [9,10] (Figure 1 and Multimedia Appendix 1). The HHIE-S questionnaire was developed by the American Speech-Language-Hearing Association (ASHA). Each question was scored as yes (4 points), sometimes (2 points), or no (0 points). The possible scores ranged from 0 (no handicap) to 40 (maximum handicap). The higher the HHIE-S scores, the greater was the handicapping effect of the hearing loss. The questionnaire takes 5 minutes to complete. Patients with scores of 10 or above were considered to have screened positive for moderate hearing loss [9].

#### Free-Field Voice Test

**Figure 2:**
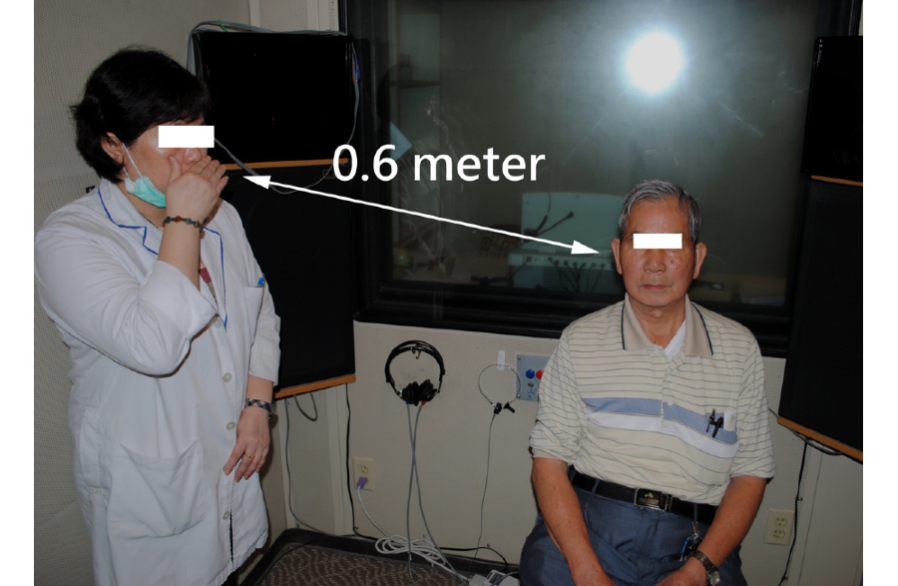
Demonstration of the free-field voice hearing screening test.

This test was performed using a sound level meter to ensure that the whispered voice corresponded to a sound level of 30-45 dB, conversational voice to a sound level of 45-60 dB, and loud voice to a sound level of 60-80 dB [6]. The examiner was asked to practice her voice levels and compare it with the sound level meter every day to ensure that her whispered voice level, conversational voice level, and loud voice level were consistent. In this test, the examiner stands 0.6 meter away from the seated patient and whispers a combination of number and letters (eg, 2-R-9 ) and asks the patient to repeat the sequence [11] (Figure 2). If the patient repeats correctly, hearing is considered normal; if the patient repeats incorrectly, the test is repeated using a different number/letter combination. The patient is considered to have passed the test if he/she repeated at least 3 out of the possible 6 numbers or letters correctly. If the patient did not pass the whispered voice test, the patient was considered to have mild hearing impairment and the test was repeated with a conversational voice. If the patient could not pass the conversational voice test, the patient was considered to have moderate hearing impairment and the test was repeated with a loud voice. The patients were divided into 3 groups in the free-field voice test; understanding the whispered voice equated with normal hearing (25-dB threshold), understanding the conversational voice level (25-40 dB threshold) was considered as mild hearing impairment, and understanding loud voice levels (41-60 dB threshold) was considered as moderate or severe (61-80 dB threshold) hearing impairment. Patients with no response to any of these voice levels (>80 dB threshold) were considered to have profound hearing impairment [6,9,11-13]. Free-field voice test was performed by the same audiologist in the same soundproof room. The patient was asked to mask his/her nontest ear during the examination. We compared the results of the free-field voice test to those of the standard pure-tone audiometry in the better-hearing ear.

#### Smartphone-Based Audiometry

**Figure 3:**
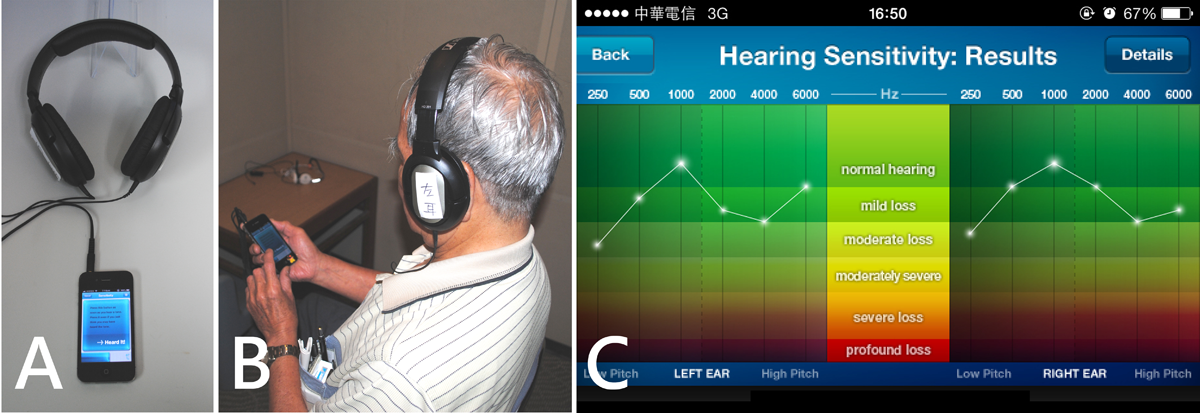
Smartphone-based audiometry hearing screening test. A: Smartphone-based audiometry device. B: Demonstration of smartphone-based audiometry. C: Results of hearing sensitivity shown in a typical audiogram format.

An iPhone 4S (Apple Inc) was used in our study. In order to avoid any possible effects of smartphone capabilities, Wi-Fi and 3G connectivity were turned off during the test. A free hearing app (uHear) was downloaded in the iPhone 4S from the iTune app store. The software enables patients to test their pure-tone air conduction hearing sensitivity. The software employs a simple “10 dB down and 5 dB up” approach [5,14]. The uHear app has the ability to determines the air-conducted sound at 250, 500, 1000, 2000, 4000, and 6000 Hz in both the left and right ears. The lowest threshold with 2 positive responses of 3 excursions was recorded as the hearing test. The hearing test can be completed in 6 minutes. Completing the hearing test required no learning curve [5,14]. At the end of the test, hearing sensitivity was shown in a typical audiogram format (Figure 3). The self-administered smartphone-based audiometry test was done in a soundproof room with an average ambient noise level of less than 35 dB hearing loss. Sennheiser HD201 headphones were used for all the patients. The patient was asked to press a large button on the touch screen to indicate when a sound was heard. Verbal instructions for the self-administered smartphone-based audiometry were presented by the same audiologist at the beginning of the test [5]. Smartphone-based audiometry test was performed by the patient in the same soundproof room. We compared the results of the smartphone-based audiometry test and those of the standard pure-tone audiometry in the better-hearing ear.

#### Standard Pure-Tone Audiometry

The Grason-Stadler GSI-61 Clinical Audiometer (AIC Medical Audiometric Instruments Corporation) and the Telephonics TDH-50P Audiometric Headphones were used to perform standard pure-tone audiometry. All clinical audiometers and accessory devices were calibrated as per ANSI S3.6, 1996 [15]. The standard pure-tone audiometry test was performed by the same audiologist in the same soundproof room. The degree of hearing loss was defined as the hearing thresholds in the better ear of the patients.

### Data Analysis

Moderate hearing impairment was defined as the mean of the hearing thresholds at 500, 1000, 2000, and 4000 Hz >40 dB hearing loss (pure-tone average [PTA]>40 dB hearing loss). The results of the hearing tests were entered into 2 × 2 tables. Sensitivity, specificity, negative likelihood ratio, and the positive likelihood ratio were calculated.

## Results

### Demographic Data of the Patients

In our study, 41 patients were recruited, of which 27 were men and 14 were women. The mean (SD) age of the patients was 72.32 (6.81) years. The mean (SD) hearing thresholds at 500, 1000, 2000, and 4000 Hz of standard pure-tone audiometry was 36.29 (15.57) dB. Table 1 summarizes the demographic characteristics of the patients.

**Table 1:** Demographic data of the patients (N=41).

Characteristics of the patients		Values
**Age (years)**		
	66-70, n (%)	22 (54)
	71-75, n (%)	7 (17)
	76-80, n (%)	6 (15)
	81-85, n (%)	3 (7)
	86-90, n (%)	3 (7)
	Mean (SD) age	72.32 (6.81)
**Gender, n (%)**		
	Male	27 (66)
	Female	14 (34)
**Better ear with pure-tone audiometry (dB)**		
	≤25, n (%)	12 (29)
	26-40, n (%)	17 (42)
	41-55, n (%)	6 (15)
	56-70, n (%)	2 (5)
	71-90, n (%)	3 (7)
	≥91, n (%)	1 (2)
	Standard pure-tone audiometry, mean (SD)	36.29 (15.57)
	Smartphone-based audiometry, mean (SD)	43.4 (15.75)

### Self-Perception Hearing Screening Test Findings

Table 2 compares the results of the self-perception hearing screening test and those of standard pure-tone audiometry. Of the 12 patients with moderate hearing impairment in the better ear (PTA>40 dB hearing loss) documented on standard pure-tone audiometry, 7 had a PTA>40 dB in the self-perception hearing screening test. This translates to a sensitivity of 0.58 (95% CI 0.29-0.84). Of the 29 patients without moderate hearing impairment in the better ear (PTA≤40 dB hearing loss) documented on standard pure-tone audiometry, 10 had a PTA≤40 dB in the self-perception hearing screening test. This translates to a specificity of 0.34 (95% CI 0.19-0.54). The positive likelihood ratio was 0.89 (95% CI 0.52-1.54). The negative likelihood ratio was 1.21 (95% CI 0.54-2.68).

**Table 2:** Accuracy of self-perception as a screening test compared to the standard pure-tone audiometry (N=41).

Self-perception test	Standard pure-tone audiometry	
	Patients with PTA^a^≤40 dB (n)	Patients with PTA>40 dB (n)
Patients with PTA≤40 dB (n)	10	5
Patients with PTA>40 dB (n)	19	7

^a^PTA: pure-tone average.

### HHIE-S Questionnaire Results

Table 3 compares the results of the HHIE-S questionnaire hearing screening test and those of standard pure-tone audiometry. Of the 12 patients with moderate hearing impairment in better ear (PTA>40 dB hearing loss) documented on standard pure-tone audiometry, 8 had a PTA>40 dB in the HHIE-S questionnaire hearing screening test. This translates to a sensitivity of 0.67 (95% CI 0.35-0.89). Of the 29 patient without moderate hearing impairment in the better ear (PTA≤40 dB hearing loss) documented on standard pure-tone audiometry, 9 had a PTA≤0 dB in the HHIE-S questionnaire hearing screening test. This translates to a specificity of 0.31 (95% CI 0.316-0.51). The positive likelihood ratio was 0.97 (95% CI 0.60-1.54). The negative likelihood ratio was 1.07 (95% CI 0.42-2.78).

**Table 3:** Accuracy of the HHIE-S questionnaire as a screening test compared to the standard pure-tone audiometry (N=41).

HHIE-S questionnaire	Standard pure-tone audiometry	
	Patients with PTA^a^≤40 dB (n)	Patients with PTA>40 dB (n)
Patients with PTA≤40 dB (n)	9	4
Patients with PTA>40 dB (n)	20	8

^a^PTA: pure-tone average.

### Free-Field Voice Test Findings

Table 4 compares the results of the free-field voice hearing screening test and those of standard pure-tone audiometry. Of the 12 patients with moderate hearing impairment in better ear (PTA>40 dB hearing loss) documented on standard pure-tone audiometry, 10 had a PTA>40 dB in the free-field voice hearing screening test. This translates to a sensitivity of 0.83 (95% CI 0.51-0.97). Of the 29 patients without moderate hearing impairment in better ear (PTA≤40 dB hearing loss) documented on standard pure-tone audiometry, 12 had a PTA≤40 dB in the free-field voice test hearing screening test. This translates to a specificity of 0.41 (95% CI 0.24-0.61). The positive likelihood ratio was 1.42 (95% CI 0.96-2.11). The negative likelihood ratio was 0.40 (95% CI 0.10-1.57).

**Table 4:** Accuracy of free-field voice test as a screening test compared to the standard pure-tone audiometry (N=41).

Free-field voice test	Standard pure-tone audiometry	
	Patients with PTA^a^≤40 dB (n)	Patients with PTA>40 dB (n)
Patients with PTA≤40 dB (n)	12	2
Patients with PTA>40 dB (n)	17	10

^a^PTA: pure-tone average.

### Smartphone-Based Audiometry Findings

Table 5 compares the results of the smartphone-based audiometry hearing screening test and those of standard pure-tone audiometry. Of the 12 patients with moderate hearing impairment in the better ear (PTA>40 dB hearing loss) documented on standard pure-tone audiometry, 11 had a PTA>40 dB in the smartphone-based audiometry hearing screening test. This translates to a sensitivity of 0.92 (95% CI 0.60-0.99). Of the 29 patients without moderate hearing impairment in the better ear (PTA≤40 dB hearing loss) documented on standard pure-tone audiometry, 22 had a PTA≤40 dB in the smartphone-based audiometry hearing screening test. This translates to a specificity of 0.76 (95% CI 0.56-0.89). The positive likelihood ratio was 3.80 (95% CI 1.95-7.4). The negative likelihood ratio was 0.11 (95% CI 0.02-0.73).

**Table 5:** Accuracy of smartphone-based audiometry as a screening test compared to the standard pure-tone audiometry (N=41).

Smartphone-based audiometry	Standard pure-tone audiometry (n)	
	Patients with PTA^a^≤40 dB (n)	Patients with PTA>40 dB (n)
Patients with PTA≤40 dB (n)	22	1
Patients with PTA>40 dB (n)	7	11

^a^PTA: pure-tone average.

## Discussion

More than 50% of the world’s population live in low-income countries. People with hearing impairments in low-income countries account for 80% of the world’s associated population [16]. Thus, most patients with hearing impairments live in the low-income countries. However, audiology services are overlooked in low-income countries, because these countries are struggling to provide even the basic medical services in order to avoid other life-threatening diseases. There is a surge of need for audiology services in these limited-income countries, because most people in these countries have neither access to an audiologist nor any form of hearing health care. According to Fagan [16], audiology services are nonexistent in most African countries or there is only a single audiologist attending to millions of people. It is estimated that there are 4 audiologists for 100,000 people in the United States. The ratio is almost the same in the United Kingdom. In Taiwan, the data from the Mackay Medical College estimated that there is only 1 audiologist per 100,000 people. Thus, audiology services are unequally distributed across the world.

In industrialized countries, well-established audiology equipment is available for audiology practice. The huge gap in the provision of audiology services between industrialized and low-income countries can barely be filled by volunteer audiologists or by developing new hearing screening methods. Therefore, we aimed to develop some fast, easy-to-use, and reliable methods for low-cost hearing screening tests. All the different methods shown in our study for screening hearing impairments have advantages and disadvantages. Smartphone-based audiometry has higher sensitivity and higher specificity than the other screening methods for detecting moderate hearing impairment in the older adults.

The first screening method for hearing impairment in this study was the self-perception hearing screening test. Self-reported data to assess the presence of diseases or disorders have been used frequently in large-scale epidemiologic survey studies in the past [7]. Studies [17] have shown that patients have been screened for hearing loss by using self-perception screening questions, which involves asking the patient whether they feel they have hearing loss, and these questions are used in the measurements of global health. Several questions can be asked, for example, “Do you have a hearing problem now?” or “Would you say you have any difficulty hearing now?” In this paper, we recommend using the question, “Do you have a hearing problem now?” as a measure of the global health in the annual medical history forms in geriatric practices [17]. A positive response to this question is considered as a positive screening for hearing loss. A total of 26 patients thought that they had hearing loss, of which 7 were tested positive for moderate hearing impairment. This single-question self-perception hearing screening test had a sensitivity of 0.58 (95% CI 0.29-0.84) and specificity of 0.34 (95% CI 0.19-0.54), with a positive likelihood ratio of 0.89 (95% CI 0.52-1.54) and negative likelihood ratio of 1.21 (95% CI 0.54-2.68). This result implied that an older person’s self-perception of a hearing problem could not reliably indicate the presence of a hearing impairment. Compared to the results in our study, the results of Gates et al [17] had higher sensitivity (0.71, 95% CI 0.63-0.78) and higher specificity (0.72, 95% CI 0.67-0.76). Cultural views of hearing impairment and disability can affect the answer to the question “Do you have a hearing problem now?” Attitudes to hearing impairment are influenced by social behavior, economic situation, and education levels, and these factors would affect the results of the self-perception hearing screening test.

The second screening method for hearing impairment in this study was the HHIE-S questionnaire hearing screening test. The screening version of the HHIE-S questionnaire is the most widely applied test that is validated. The HHIE-S questionnaire is a 10-item, self-administered questionnaire that was developed to measure the emotional and social handicap [7]. The ASHA-suggested fail-criteria of 10 points or more equals to moderate hearing impairment. A total of 28 patients scored more than 10 points in the HHIE-S questionnaire, of which 8 tested positive for moderate hearing impairment. This HHIE-S questionnaire hearing screening test had a sensitivity of 0.67 (95% CI 0.35-0.89) and specificity of 0.31 (95% CI 0.316-0.51), with a positive likelihood ratio of 0.97 (95% CI 0.60-1.54) and negative likelihood ratio of 1.07 (95% CI 0.42-2.78). Yueh et al showed that the sensitivity of the HHIE-S questionnaire hearing screening test ranges from 0.53 to 0.80 and the specificity ranges from 0.67 to 0.75 [18]. The sensitivity of the single-question self-perception hearing screening test was only 0.58 in our study. The 10-item HHIE-S questionnaire hearing screening test, which is related to daily activities, includes only a single question, which increases the sensitivity of the HHIE-S questionnaire to 0.67. Both single-question self-perception and the 10-item HHIE-S questionnaire hearing screening tests are used for assessing the subjective hearing status. However, our results suggest that these tests may be less effective in screening early stages of hearing impairment.

The use of speech to determine hearing impairment level has been used for a long time. The hearing impairment level can be assessed by a primary health care doctor by using the free-field voice hearing screening test. It is surprising that the free-field voice hearing screening test is still performed nowadays. In an industrialized country like United Kingdom, primary health care doctors are obliged to screen the older adult population for hearing impairment, as listed in the 1990 National Health Service contract [19]. The primary health doctor uses the free-field voice hearing screening test as the first examination for hearing impairment in the United Kingdom. We used a sound level meter to calibrate the voice of the examiner. In order to ensure that the whispered voice level, conversational voice level, and loud voice level were consistent, the examiner was asked to calibrate her voice levels every day. A total of 27 patients were considered to have moderate hearing impairment in the free-field voice hearing screening test, of which 10 were tested positive for moderate hearing impairment. This free-field voice hearing screening test had a sensitivity of 0.83 (95% CI 0.51-0.97) and specificity of 0.41 (95% CI 0.24-0.61), with a positive likelihood ratio of 1.42 (95% CI 0.96-2.11) and negative likelihood ratio of 0.40 (95% CI 0.10-1.57). McShefferty et al [20] showed that the sensitivity of the free-field voice hearing screening test was 0.56 and that the specificity was 0.65. The results of our study showed higher sensitivity and lower specificity. The potential reason for our findings could be explained by the calibration of the sound level, which resulted in higher sensitivity.

In recent years, there has been a rapid evolution in the development of mobile health services. Smartphone-based audiometry is considered as a fast, easy, and reliable technique for cost-effective screening of hearing impairments. Free apps can be downloaded from the Android market and installed in any smartphone. Smartphones have the ability to control the audio output and frequency. However, given that there are difficulties with calibration, in terms of sound output levels and timing uncertainty, the applicability of smartphones and standard pure-tone audiometry is limited. A total of 18 patients were considered to have moderate hearing impairment in the smartphone-based audiometry hearing screening test, of which 11 tested positive for moderate hearing impairment. The smartphone-based audiometry hearing screening test had a sensitivity of 0.92 (95% CI 0.60-0.99) and specificity of 0.76 (95% CI 0.56-0.89), with a positive likelihood ratio of 3.80 (95% CI 1.95-7.4) and negative likelihood ratio of 0.11 (95% CI 0.02-0.73). Szudek et al [5] showed that the sensitivity of smartphone-based audiometry was 0.98 and the specificity was 0.82. The results of our study showed lower sensitivity and specificity compared to those reported in previous studies [21-27], probably because our study population consisted of adults older than 65 years. Older populations may not be good at operating a smartphone. Despite the above limitations, our study shows that smartphone-based audiometry is a reasonable hearing test for diagnosing moderate hearing impairment. Smartphone-based audiometry could be the next trend for screening hearing impairment in older adults, especially in low-income countries. Our study specifically excluded people with hearing aids or those who received standard pure-tone audiogram evaluation in the prior 24 months, because the purpose of this study was to detect older adults with unrecognized moderate hearing impairment.

In conclusion, all the different methods mentioned in this study are available for hearing screening but they all have advantages and disadvantages. Smartphone-based audiometry has higher sensitivity and higher specificity for detecting moderate hearing impairment in older populations. Prospective randomized clinical studies are still needed to test the application of smartphone-based audiometry in the field of screening for hearing impairment in older populations. Nevertheless, we recommend that in low-income countries where specialized audiological services and proven audiometric equipment are not available, primary health care givers should be trained to administer simple smartphone-based audiometry as an acceptable alternative hearing assessment for older adults who have complaints with regard to hearing impairment.

## Appendix

Multimedia Appendix 1 Hearing Handicap Inventory for the Elderly-Screening Questionnaire in Chinese.

## Abbreviations

ASHA: American Speech-Language-Hearing Association
HHIE-S: Hearing Handicap Inventory for the Elderly-Screening
PTA: pure-tone average
