# Supplementary figures and images for: Screening for Hearing Impairment in Older Adults by Smartphone-Based Audiometry, Self-Perception, HHIE Screening Questionnaire, and Free-Field Voice Test: Comparative Evaluation of the Screening Accuracy With Standard Pure-Tone Audiometry
Source: JMIR Mhealth Uhealth. 2020 Oct 27;8(10):e17213. doi: 10.2196/17213 (PMC7655471; doi:10.2196/17213)

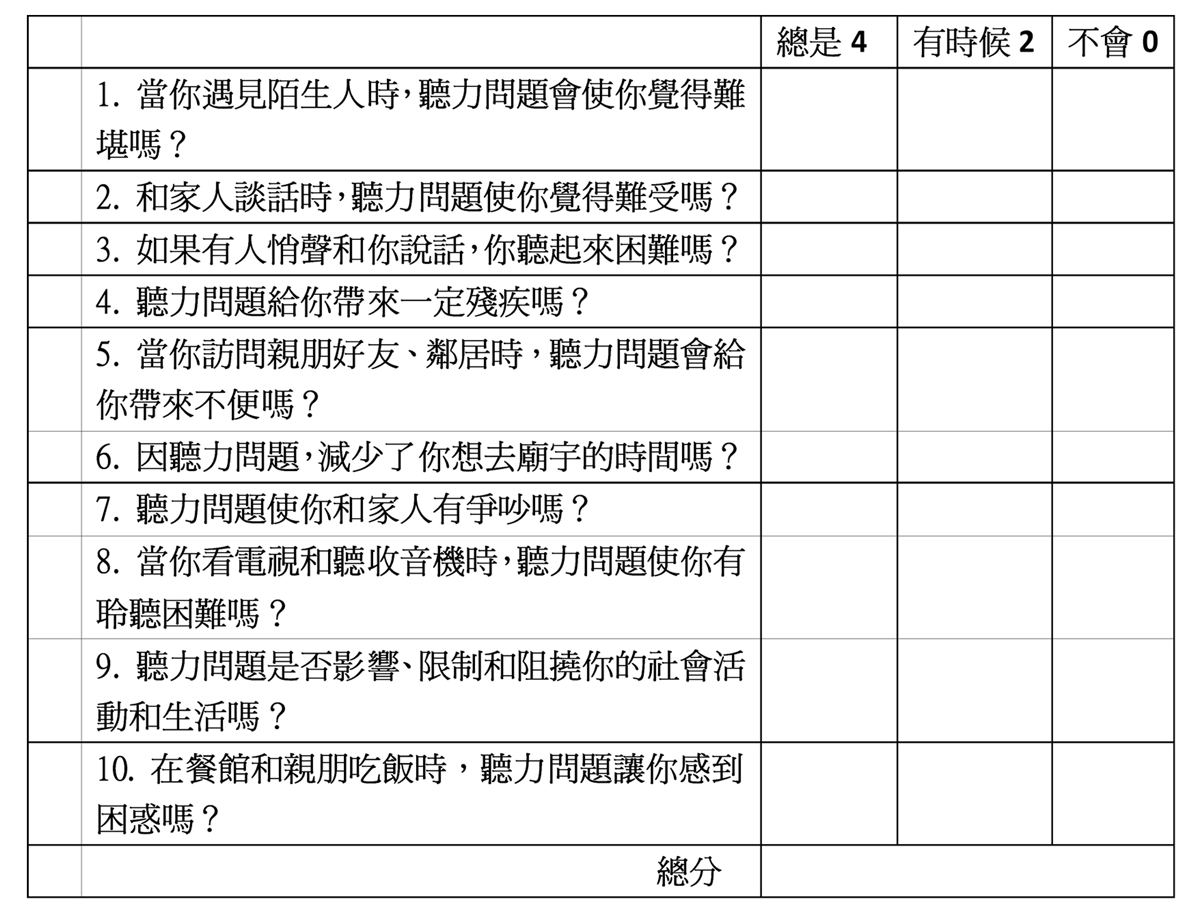

Supplement: Multimedia Appendix 1 [file mhealth_v8i10e17213_app1.png]
